# PD-1 Blockade and OX40 Triggering Synergistically Protects against Tumor Growth in a Murine Model of Ovarian Cancer

**DOI:** 10.1371/journal.pone.0089350

**Published:** 2014-02-27

**Authors:** Zhiqiang Guo, Xin Wang, Dali Cheng, Zhijun Xia, Meng Luan, Shulan Zhang

**Affiliations:** 1 Department of Gynecology and Obstetrics, Shengjing Hospital, China Medical University, ShenYang, China; 2 Department of Gynecology and Obstetrics, No. 306 Hospital of PLA, Beijing, China; 3 Department of Gynecology and Obstetrics, The First Affiliated Hospital, China Medical University, Shen Yang, China; Mie University Graduate School of Medicine, Japan

## Abstract

The co-inhibitory receptor Programmed Death-1 (PD-1) curtails immune responses and prevent autoimmunity, however, tumors exploit this pathway to escape from immune destruction. The co-stimulatory receptor OX40 is upregulated on T cells following activation and increases their clonal expansion, survival and cytokine production when engaged. Although antagonistic anti-PD-1 or agonistic anti-OX40 antibodies can promote the rejection of several murine tumors, some poorly immunogenic tumors were refractory to this treatment. In the present study, we evaluated the antitumor effects and mechanisms of combinatorial PD-1 blockade and OX40 triggering in a murine ID8 ovarian cancer model. Although individual anti-PD-1 or OX40 mAb treatment was ineffective in tumor protection against 10-day established ID8 tumor, combined anti-PD-1/OX40 mAb treatment markedly inhibited tumor outgrowth with 60% of mice tumor free 90 days after tumor inoculation. Tumor protection was associated with a systemic immune response with memory and antigen specificity and required CD4^+^ cells and CD8^+^ T cells. The anti-PD-1/OX40 mAb treatment increased CD4^+^ and CD8^+^ cells and decreased immunosuppressive CD4^+^FoxP3^+^ regulatory T (Treg) cells and CD11b^+^Gr-1^+^ myeloid suppressor cells (MDSC), giving rise to significantly higher ratios of both effector CD4^+^ and CD8^+^ cells to Treg and MDSC in peritoneal cavity; Quantitative RT-PCR data further demonstrated the induction of a local immunostimulatory milieu by anti-PD-1/OX40 mAb treatment. The splenic CD8^+^ T cells from combined mAb treated mice produced high levels of IFN-γ upon tumor antigen stimulation and exhibited antigen-specific cytolytic activity. To our knowledge, this is the first study testing the antitumor effects of combined anti-PD-1/OX40 mAb in a murine ovarian cancer model, and our results provide a rationale for clinical trials evaluating ovarian cancer immunotherapy using this combination of mAb.

## Background

Ovarian carcinoma (OC) is the most lethal malignancy in women, with 22,280 new cases and 15,460 deaths estimated in the United States for 2012 [1]. The high rate of lethality from OC is primarily due to the advanced stage of disease at diagnosis. Early stage cancers can be cured in up to 90% of patients with current therapies [2], but this rate drops substantially for advanced disease with approximately 30% of patients with advanced stage OC survive 5 years after initial diagnosis [3]. The standard treatment for ovarian cancer is surgical debulking followed by platinum-taxane based chemotherapy [4]. Although most patients are responsive to chemotherapy at first, the majority of them will eventually have a relapse and die of the disease. Therefore, novel strategies are urgently needed to improve the outcomes of ovarian cancer.

Accumulating evidence suggests that immunotherapy should be effective for OC treatment [5]. Firstly, OC cells express many tumor-associated antigens against which specific immune responses have been detected [6]–[10]. Secondly, the studies pioneered by Coukos and colleagues indicate tumor immune response is a critical determinant of clinical outcomes of patients with OC supported by the close correlation between survival of these patients and tumor infiltration with CD3^+^ T cells in the large annotated clinical samples [11]. Thirdly, although OC is a devastating disease, metastases are frequently restricted to the peritoneal cavity where the tumor microenvironment is directly accessible, which obviates the need for systemic delivery of immunostimulatory treatments [12]. Despite the abundant evidence supporting OC immunotherapy, clinical success with immune-based therapies for OC has generally been modest [13].

Programmed Death 1 (PD-1) protein is a key coinhibitory receptor on T cells with a structure similar to that of CTLA-4 but with a distinct biologic function and ligand specificity [14]. PD-1 functions primarily in peripheral tissues, where T cells may encounter the immunosuppressive PD-1 ligands PD-L1 (B7-H1) and PD-L2 (B7-DC), which are expressed by tumor cells, stromal cells, or both [15], [16]. Blockade of the interaction between PD-1 and PD-L1 potentiates T-cell immune responses in vitro and mediates preclinical antitumor activity [16]–[18]. PD-L1 is the primary PD-1 ligand that is up-regulated in solid tumors, where it can inhibit cytokine production and the cytolytic activity of PD-1^+^ tumor-infiltrating CD4^+^ and CD8^+^ T cells [14], [19]. These features make PD-1/PD-L1 pathway a promising intervention target for tumor immunotherapy, which is validated by the recently reported results from two clinical trials showing mAbs specific for PD-1 and PD-L1 trigger an impressive antitumor effect in non-small cell lung cancer, melanoma and renal-cell cancer with complete regression achieved in some patients [20]–[22].

OX40 (a.k.a CD137) is a costimulatory molecule belonging to the TNF receptor family expressed primarily on activated effector T (T eff) cells and naive regulatory T cells [23]. Ligation of OX40, primarily on CD4^+^ T cells, activates NF-κB pathway and up-regulates antiapoptotic molecules of the Bcl-2 family, leading to T cell clonal expansion, activation, memory, and cytokine production [24]–[26]. OX40 engagement on CD4^+^ FoxP3^+^ Treg cells leads to expansion, deactivation, or cell death depending on the local milieu [27]–[30]. Given that OX40 triggering can potently stimulate T cells and potentially block/eliminate regulatory T cells, OX40 agonists have been investigated in multiple preclinical tumor models [31]–[34] and an anti-human OX40 monoclonal antibody is currently being evaluated in clinical trials (Clinical trial registration numbers NCT01303705, NCT01416844, NCT01862900, NCT01644968 and NCT01642290).

Although antagonist PD-1 or agonistic OX40 antibodies can promote the rejection of some murine tumors, however, poorly immunogenic tumors such as ID8 ovarian cancer do not respond to antibody therapy alone [35]. We hypothesized that combined PD-1 blockade and OX40 activation would promote the antitumor immunity. Here, using ID8 murine ovarian cancer model, we show that combined anti-PD-1/OX40 mAb treatment significantly suppressed the 10 days established peritoneal ID8 tumor growth, resulting in 60% of treated mice tumor free 90 days after tumor injection. Closer examination revealed that combined anti-PD-1/OX40 mAb prominently increased the effector T cells and attenuated the immunosuppressive cells in tumor sites with the induction of systemic antigen-specific CTL response.

## Methods

### Mice

Female C57BL (6-8 wk old) were purchased from the Animal Experimental Center of the China Medical University. Animal use was approved by the Institutional Review Board of China Medical University

### Cell Culture

ID8, a clone of the MOSEC ovarian carcinoma of C57BL/6 origin was a gift from Dr. George Coukos (University of Pennsylvania, Philadelphia, USA) [36]. Murine B16 melanoma cells, TC1 lung cancer and T cell lymphoma EL4 cells were purchased from ATCC (Manassas, VA). Tumor cells were cultured in the complete DMEM medium supplemented with 10% FBS (Thermo Scientific, Rockford, IL), 100 U/mL penicillin and 100 µg/mL streptomycin before cell suspensions were prepared and transplanted to mice. The EL4 cells and splenocytes were maintained in a complete medium of RPMI-1640 supplemented with 10% FBS, 25 mM HEPES, 2 mM glutamine, 100 U/mL penicillin and 100 µg/mL streptomycin.

### Monoclonal Antibodies (mAbs)

Therapeutic anti-OX40 (Clone OX-86; Catalog#:BE0031), anti-PD-1 (Clone RMT3-23; Catalog#BE0115), anti-CD4 (Clone GK1.5; Catalog#:BE0003-1), anti-CD8 (Clone 2.43; Catalog#:BE0061), anti-NK1.1 (Clone PK136; Catalog#:BE0036) and control rat IgG2a mAb (Clone 2A3; Catalog#:BE0089) were purchased from BioXcell (West Lebanon, NH). Antibodies used for flow cytometry were purchased from Tianjing Sungene (Tianjing, China) and eBioscience (San Diego, CA).

### Animal experiments

Mice (5 or 10 mice/group) were injected intraperitoneally (i.p.) with 1×10^6^ ID8 cells in 0.1 mL of PBS. At days 10, 14 and 18 after tumor inoculation, each mouse received the i.p. injection of 200 µg of control, anti-PD-1, anti-OX40 or anti-PD-1/OX40 mAb in 200 µL of PBS. The mice were weighed twice weekly and checked daily for the clinical sign of swollen bellies indicative of ascites formation and for the evidence of toxicity such as respiratory distress, mobility, weight loss, diarrhea, hunched posture, and failure to eat while histopathology was conducted on major organs (i.e., liver, kidney, intestines, lungs, and colon). Following institutional guidelines, mice were killed when they developed ascites and had a weight increase >30%. The peritoneal tumor masses were collected and weighed, the survival of each mouse was recorded and overall survival was calculated.

For immune memory evaluation, pooled (2 independent experiment) 12 long-term surviving mice (90 days after first tumor injection) from combined anti-PD-1/OX40 therapy group or age-matched naïve mice (which served as control) were challenged i.p. or subcutaneously (s.c.) with 1×10^6^ ID8 cells or 1×10^6^ syngeneic but antigenically different TC1 cells. Three perpendicular diameters of s.c. tumors were measured every second day using a caliper and tumor volumes were calculated according to the formula: 1/2× (length) × (width)^2^. Mice were sacrificed when they seemed moribund or their tumors reached 10 mm in diameter. The survival of each mouse was recorded and overall survival was calculated.

For depletion of lymphocyte subsets, mice were injected i.p. with 500 µg of mAb against CD8, CD4, or NK1.1, 1 day before and two days after tumor challenge, followed by injection of 200 µg every 5 days throughout the experiment. The efficacy of cell depletion was verified by staining peripheral blood leukocytes for specific subsets after depletion (data not shown).

### Evaluation of peritoneal immune cells (PIC)

Mice which had been transplanted i.p. with ID8 cells were euthanized 7 days after they had been treated with control, single or combined mAb combination described as in animal experiments. To obtain PIC, 3 ml PBS was injected into the peritoneal cavity of mice with ID8 tumors immediately after euthanasia, their belly was massaged and the fluid was removed, filtered through a 70 µM cell strainer (BD Biosciences), washed and immune cells were isolated by using a mouse lymphocyte isolation buffer (Cedarlane, Burlington, Ontario) following the manufacturer's instruction and most of resultant cells (>95%) were CD45-postive immune cells as confirmed by flow cytometric analysis (Figure S1).

For flow cytometric staining, single cell suspensions of PIC were washed with FACS staining buffer and incubated with mouse Fc receptor binding inhibitor (eBioscience) for 10 minutes before staining with mAbs (Tianjing Sungene) against mouse CD45 (clone 30-F11), CD3 (clone 145-2C11), CD4 (clone GK1.5), CD8 (clone 53-6.7), CD19 (clone eBio1D3), CD11b (clone M1/70) and Gr-1 (clone RB6-8C5), CD44 (clone 1M7) and CD62L (clone MEL-14) for 30 minutes. For intracellular staining of FoxP3 (clone FJK-16 s; eBioscience), cells were fixed, permeabilized and stained following the instruction of Cytofix/Cytoperm kit (BD Bioscience). Flow cytometry was performed using FACSCalibur (BD Biosciences) and the population of immune cells was selected by gating CD45-positive cells. The data were analyzed using Flow Jo software (Tree Star, Ashland, OR). All flow cytometry experiments were performed at least 3 times.

### Quantitative RT-PCR

Total cellular RNA was extracted using RNeasy Mini Kits (Qiagen, Hilden, GA) and reverse transcribed into cDNA using SuperScript III Reverse Transcriptase (Invitrogen). Expression for genes of interest was analyzed in PIC on day 7 after the third injection of mAb. The primers for all genes tested, including internal control GAPDH, were synthesized by Invitrogen, Shanghai, China. Primer sequences were as follows:GAPDH:For:5′-GTGGAGATTGTTGCCATCAACG-3′,Rev:5′-CAGTGGATGCAGGGATGATGTTCTG-3′;T-bet:For:5′-CGGTACCAGAGCGGCAAGT-3′, Rev:5′-AGCCCCCTTGTTGTTGGTG-3′; FoxP3:For:5′-CAGCTGCCTACAGTGCCCCTAG-3′, Rev:5′-CATTTGCCAGCAGTGGGTAG-3′; IFN-γ: For: 5′-AAAAACCTAAAAAATCTAAATAACT-3′, Rev: 5′-ATCAACAACAACTCCTTTTCCACTT-3′; IL-10:For:5′-CTCTTACTGACTGGCATGAGG-3′,Rev:5′-CCTTGTAGACACCTTGGTCTTGGAG-3′. Quantitative real-time PCR was performed via ABI PRISM 7500 Real-Time PCR Systerm (Applied Biosystems) with 1× SYBR Green Universal PCR Mastermix (Takara). Transcript levels were calculated according to the 2–ΔΔCt method, normalized to the expression of GAPDH, and expressed as fold change compared with control.

### Analysis of IFN-γ and IL-10 production by PIC

Mice injected i.p. with 1×10^6^ ID8 cells 10 day earlier were injected thrice at 4 days interval with 200 µg of control or anti-PD-1/OX40 mAb. Seven days after the last mAb injection, pooled PIC (2×10^6^/well) harvested from treated mice were then stimulated with 50 ng/ml PMA and 1 µg/ml ionomycin for 6 hours prior to the analysis of IL-10 and IFN-γ secretion in culture supernatants by Mouse IFN-γ/IL-10 Quantikine ELISA Kit according to the manual (R&D systems, Minneapolis, MN).

### Evaluation of antigen-specific immune response

Isolated splenocytes from treated mice were cultured in the presence of 10 µg/mL H-2Db-restricted mesothelin-derived peptides (amino acid 406-414) or control HPV-E7-derived peptide (amino acid 49-57; all from GenScript, Nanjing, CA) for 3 days. IFN-γ in the supernatants was determined by Mouse IFN-γ Quantikine ELISA Kit (R&D systems). The results were also analyzed after normalization to the percentages of splenic CD8^+^ T cells.

For mesothelin-specific CTL assays, effector cells were obtained by coculturing 5×10^6^ splenocytes with 5×10^5^ UV-irradiated ID8 cells for 4 days. Peptide-pulsed EL4 target cells were generated by adding 10 µg/ml of peptide and incubating for 4 hours. CTL activity was measured using the CytoTox96 Non-Radioactive Cytotoxicity Assay kit (Promega, Madison, WI) following the manufacturer's instructions. In brief, target cells were incubated with varying numbers of effector cells for about 4 hours, and supernatants were then analyzed for lactate dehydrogenase release. The results are expressed as percent specific lysis, calculated as (Experimental release-Spontaneous release/Total release-Spontaneous release) ×100. In some experiments, effector cells were incubated with anti-CD4 or CD8 antibody (10 µg/mL) for 2 hours before CTL assay.

For mesothelin-specific antibody evaluation, we applied the method described previously [37]. Blood was obtained from naive mice (5 mice) or single mAb treated mice when they euthanized (5 mice) or 2 mAb treated long-term survivors (90 days after the tumor inoculation; 4 mice). The presence of mesothelin-specific antibodies was determined by staining the mouse ID8 ovarian cancer cells using serum from treated mice in a 1∶200 dilution, followed by staining with secondary Phycoerythrin (PE)-conjugated anti-mouse IgG antibody (eBioscience). Staining with commercially available mouse anti-mesothelin IgG (sc-33672; Santa Cruz Biotechnology, Dallas, Texas) plus secondary antibody or secondary antibody alone was used as positive or negative control. The ratios of mean fluorescence intensity (MFI) from positive control or sera to MFI from negative control were calculated.

### Statistics

Results were expressed as mean ± SEM. All statistical analyses were performed using GraphPad Prism 5. Student's t test was used to compare the statistical difference between two groups and one-way ANOVA was used to compare three or more groups. Survival rates were analyzed using the Kaplan–Meier method and evaluated with the log-rank test with Bonferroni correction. Significant differences were accepted at p<0.05.

## Results

### Combined anti-PD-1/OX40 mAb treatment induced a synergistic antitumor effect in ID8 tumor-bearing mice

**Figure 1:**
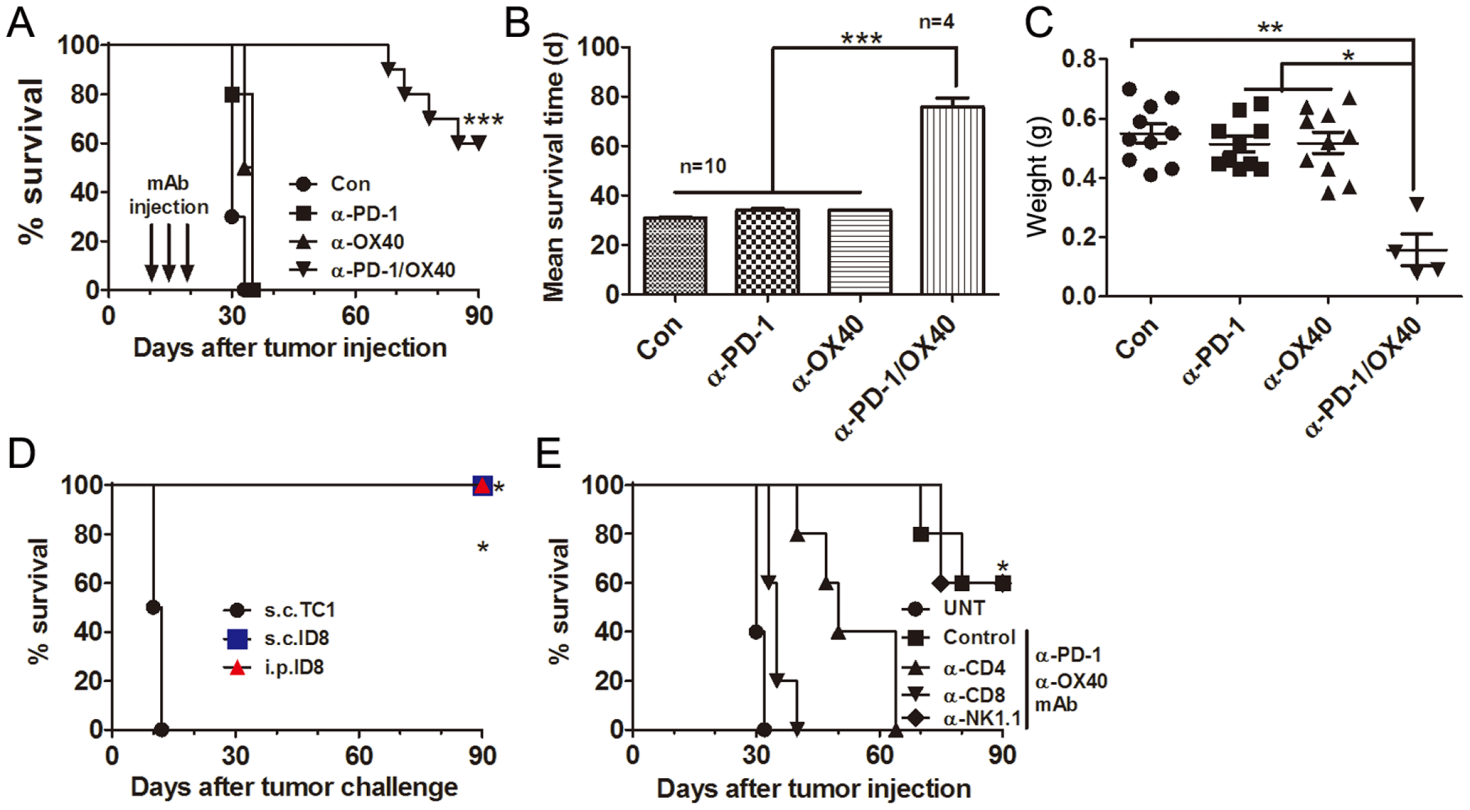
Treatment of combined anti-PD-1/OX40 mAb induced tumor-specific long-lasting immunity against ID8 ovarian cancer. A, Mice (10 mice per group) transplanted i.p. with 1×10^6^ ID8 cells 10 day before were treated thrice with 200 µg of control, anti-PD-1, anti-OX40 and anti-PD-1/OX40 mAb at 4 days interval and overall survival of mice was recorded. B, Mean survival time of mice with tumor growth was calculated. C, The peritoneal tumor masses was weighed when mice were euthanized with each dot representing each mouse. D, Long-term surviving (90 days after first tumor challenge) mice (4 mice per group) from 2 mAb treatment group were rechallenged with ID8 cells given i.p. or s.c. or with syngeneic unrelated TC1 cells transplanted s.c., and then overall survival of mice was recorded. E, Mice (5 mice per group) treated with combined anti-PD-1/OX40 mAb were also injected with an anti-CD4, anti-CD8, anti-NK1.1, or control mAb with 500 µg of each mAb per mouse 1 day before and two days after tumor challenge followed by injection of 200 µg every 5 days thereafter for the duration of the experiments. Tumor-bearing untreated mice were as negative controls (UNT). Overall survival of mice was recorded. Data are representative of three independent experiments except for D and E, which was from one experiment. ***P<0.001, combined mAb vs control or single mAb treated mice in A-C; ***P<0.001, s.c. or i.p.ID8 vs s.c.TC1 in C; * P<0.05, control or NK1.1 vs UNT, CD4 or CD8 in D.

We evaluated the antitumor efficacy of single or combined anti-PD-1 and anti-OX40 mAb in C57BL/6 mice transplanted i.p. 10 days previously with 1×10^6^ ID8 cells. Untreated mice and mice treated with a control mAb developed ascites about 30 days after tumor inoculation and had to be sacrificed as described previously [38]. As shown in Figure 1A, either single mAb had little effects on the growth of 10 days established ID8 tumor leading to ascites formation at the almost same time as untreated or control mAb treated mice; however, combined anti-PD-1/OX40 mAb treatment significantly increased overall survival of mice with 60% (6 out of 10 mice) of mice tumor free (confirmed by laparotomy) 90 days after tumor injection (p<0.001, combined mAb compared to single or control mAb), and even mice succumbed to tumor growth also had significantly prolonged mean survival time (MST) compared with control or single mAb treated mice (Figure 1B; MST 30.90, 34.00, 34.00and 75.75 days for control, anti-PD-1, anti-OX40 and anti-PD-1/OX40 group; p<0.001, combined mAb compared to single or control mAb). The weight of tumor masses from mice treated with combined mAb also greatly decreased compared with that from control or single mAb treated mice (Figure 1C). A repeat of the experiment gave similar results (data not shown). In these animal experiments, we did not observe any obvious toxicity such as weight or hair loss in mice receiving single or combined anti-PD-1/OX40 mAb. In addition, we also did not detect any expression of PD-1 and OX40 molecules and their respective ligands PD-L1/PD-L2 and OX40L on the surface of ID8 ovarian cancer cells (data not shown), excluding the possibility that inhibition of ID8 tumor growth *in vivo* is directly mediated by anti-PD-1 or anti-OX40 mAb.

Mice rejecting ID8 cells after combined treatment were resistant to a subsequent rechallenge i.p. or s.c. with the same cell line but not s.c. with unrelated TC1 lung cancer cells (Figure 1D). These results indicate that combined treatment induces a specific and long-lasting antitumor immune response in treated mice. Lymphocyte subset depletion experiments demonstrated that tumor protection by anti-PD-1/OX40 mAbs was dependent on the CD4^+^ and CD8^+^ T cells as removal of CD4^+^ or CD8^+^ T cells but not NK cells completely abrogated the antitumor effect conferred by anti-PD-1/OX40 mAb treatment (Figure 1E).

### Combined anti-PD-1/OX40 mAb treatment significantly increases ratios of both CD4 and CD8 T cells to Treg and MDSC

**Figure 2:**
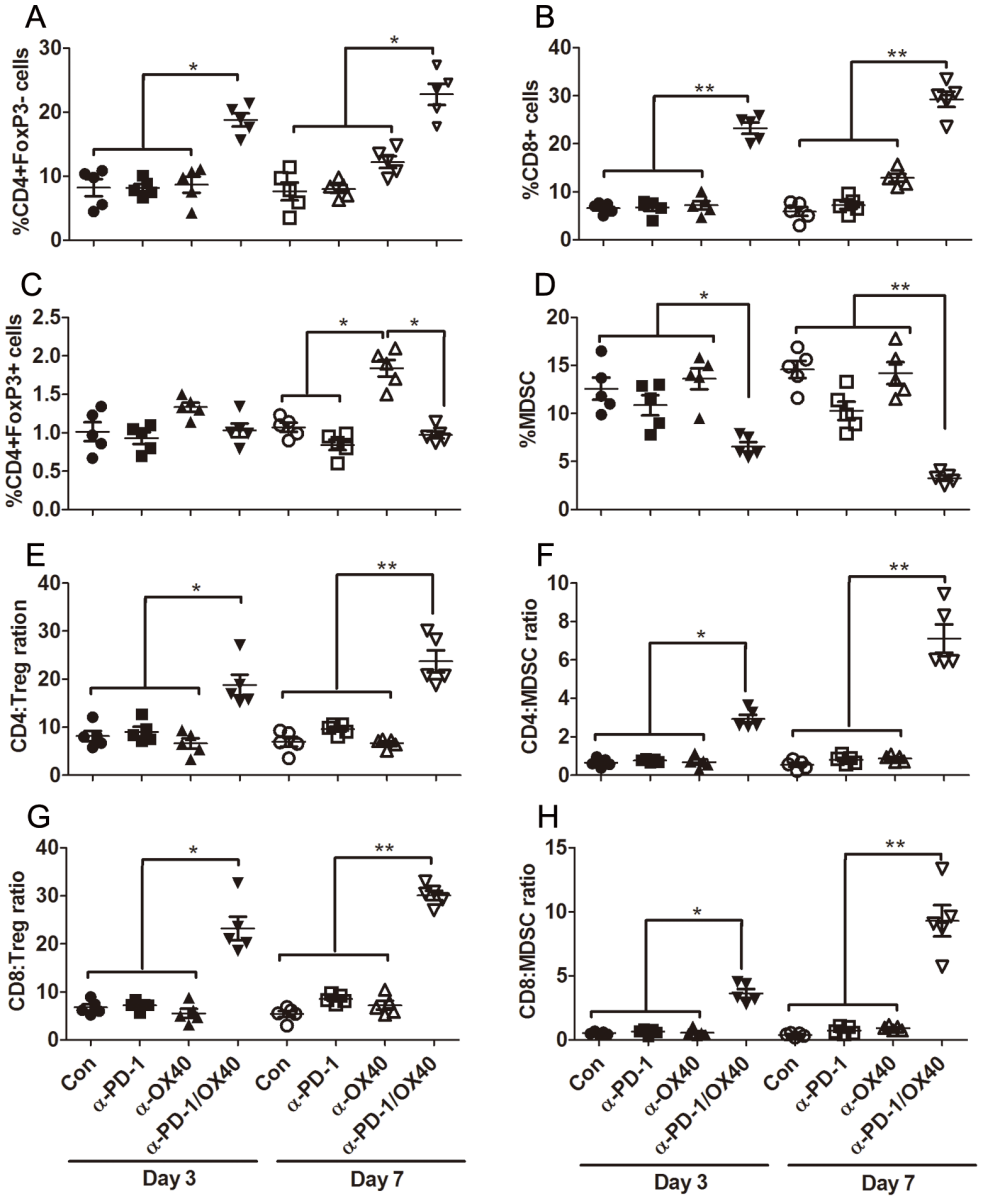
Composition analysis of peritoneal immune cells (PIC) from treated mice. Mice (5 mice per group) injected i.p. with 1×10^6^ ID8 cells 10 day earlier were injected thrice at 4 days interval with 200 µg of control, single or combined anti-PD-1/OX40 mAb. Three or seven days later, peritoneal lavages from treated mice were analyzed by flow cytometry for the composition of various immune subsets. The percentages of CD4^+^FoxP3^−^ T cells, CD8^+^ T cells, CD4^+^FoxP3^+^ Treg and CD11b^+^GR-1^+^ MDSC in CD45^+^ peritoneal immune cells are shown in A, B, C and D respectively with each dot representing data from each mouse. The ratios of both CD4^+^ and CD8^+^ T cells to Treg and MDSC are shown in E, F, G and H respectively with each dot representing data from each mouse. Data are representative of 2 independent experiments, *P<0.05, **P<0.01.

To explore the mechanisms underlying the synergy between PD-1 blockade and OX40 activation, we analyzed the effects of single or combined mAb on tumor-infiltrating peritoneal immune cells (PIC) harvested from treated mice 3 or 7 days after last mAb injection. Compared with control or single mAb, combined mAb significantly increased the percentages of effector CD4^+^FoxP3^-^ (mean value for control, anti-PD-1, anti-OX40 and anti-PD-1/OX40 group: 8.24%, 8.20%, 8.70%, 18.78% at day 3 and 7.66%, 8.02%, 12.22%, 22.80% at day 7; p<0.05) and CD8+ (mean value 6.62%, 6.74%, 7.20%, 23.24% at day 3 and 5.88, 7.22, 12.90, 29.26 at day 7; p<0.01) T cells and decreased the frequency of CD11b^+^GR-1^+^ myeloid-derived suppressor cells (MDSC; mean value 12.58%, 10.86%, 13.62%, 6.53% at day 3 and 14.58%,10.28%,14.20%,3.28% at day 7; p<0.05 and 0.01 for day 3 and 7 respectively) in PIC on day 3 after treatment and changes became more prominent on day 7 after treatment (Figure 2A-D); however, no alteration in the percentage of CD4^+^FoxP3^+^ regulatory T cells (Treg) was observed in combined mAb treated mice at two time points evaluated. These contrasting changes in effector and immunosuppressive cells gave rise to the significantly elevated ratios of both effector CD4^+^ and CD8^+^ T cells to Treg and MDSC in peritoneal cavity of mice receiving combined mAb treatment (Figure 2E-H). With regards to individual mAb treatment, OX40 engagement significantly elevated the percentage of CD4^+^FoxP3^+^ Treg (1.01%, 0.93%, 1.33%, 1.03% at day 3 and 1.07%, 0.84%, 1.84%, 0.97% at day 7; p<0.05 at day 7) with modest increase in the percentage of effector CD4^+^FoxP3^-^ and CD8+ T cells on day 7 after treatment; however, single PD-1 blockade mildly attenuated the levels of immunosuppressive Treg and MDSC in peritoneal cavity. We noted an increase in absolute number of total immune cells from 2 mAb treated mice at two time points analyzed (mean value (×10^6^/mouse): 3.5, 3.7, 3.8, 5.6 at day 3 and 3.7, 3.8, 4.0, 6.2 at day 7; p<0.05) and the changes in absolute number of each subset had a similar trend to their percentages (data not shown).

**Figure 3:**
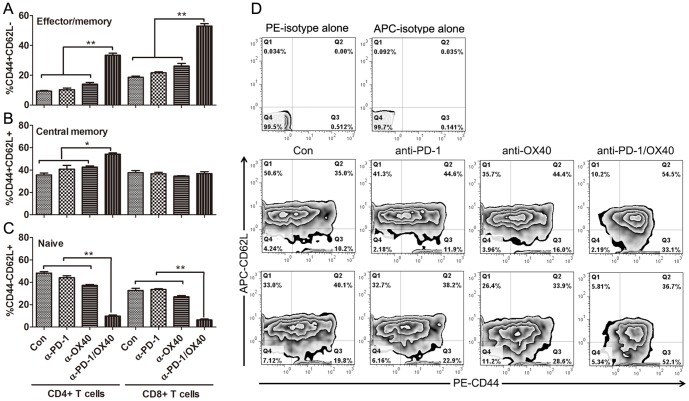
Phenotypic analysis of peritoneal CD4^+^ and CD8^+^ T cells from treated mice. Mice (5 mice per group) injected i.p. with 1×10^6^ ID8 cells 10 day earlier were injected thrice at 4 days interval with 200 µg of control, single or combined anti-PD-1/OX40 mAb. Seven days later, the expression of CD44 and CD62L on peritoneal CD4^+^ and CD8^+^ T cells from treated mice was analyzed by flow cytometry. The frequencies of CD44^+^CD62L^−^ effector/memory, CD44^+^CD62L^+^ central memory and CD44^−^CD62L^+^ naïve cells in peritoneal CD4^+^ and CD8^+^ T cells are shown in A, B and C respectively. The representative dotplots are shown in D with upper, middle and bottom panels displaying isotype, CD44 and CD62L staining in gated CD4^+^ and CD8^+^ T cells respectively. Data are representative of 2 independent experiments, *P<0.05, **P<0.01.

Further phenotype analysis demonstrated that significantly increased percentage of CD44^+^CD62L^−^ effector/memory (mean value for control, anti-PD-1, anti-OX40 and anti-PD-1/OX40 group: 9.4%, 10.3%, 14.0%, 33.4%, and 18.6%, 21.6%, 26.2%, 53.0% for CD4 and CD8 cells; p<0.01 for both cells) and CD44^+^CD62L^+^ central memory (35.%, 40.9%, 42.8%, 54.2%, and 37.7%, 36.7%, 34.6%, 36.9% for CD4 and CD8 cells; p<0.05 for CD4 cells) cells was seen in peritoneal CD4+ and CD8+ T cells from anti-PD-1/OX40 mAb treated mice compared with that from control or single mAb treated mice (Figure 3A, B), whose PIC contained higher levels of naïve CD4^+^ and CD8^+^ T cells (48.2%, 44.1%,37.2%,9.73%, and 32.7%, 33.6%, 27.1%, 6.3% for CD4 and CD8 cells; p<0.01 for both cells) with CD44^−^CD62L^+^ phenotype (Figure 3C). The representative dotplots were shown in Figure 3D.

In summary, these results indicate that concomitant PD-1 blockade and OX40 triggering creates higher ratios of effector T cells to immunosuppressive cells in peritoneal cavity of treated mice, which represents the shift of the normally suppressive tumor milieu to a more stimulatory state which is more permissive for immune mediated tumor destruction.

### Combined anti-PD-1/OX40 mAb treatment fostered a local immunostimulatory microenvironment

**Figure 4:**
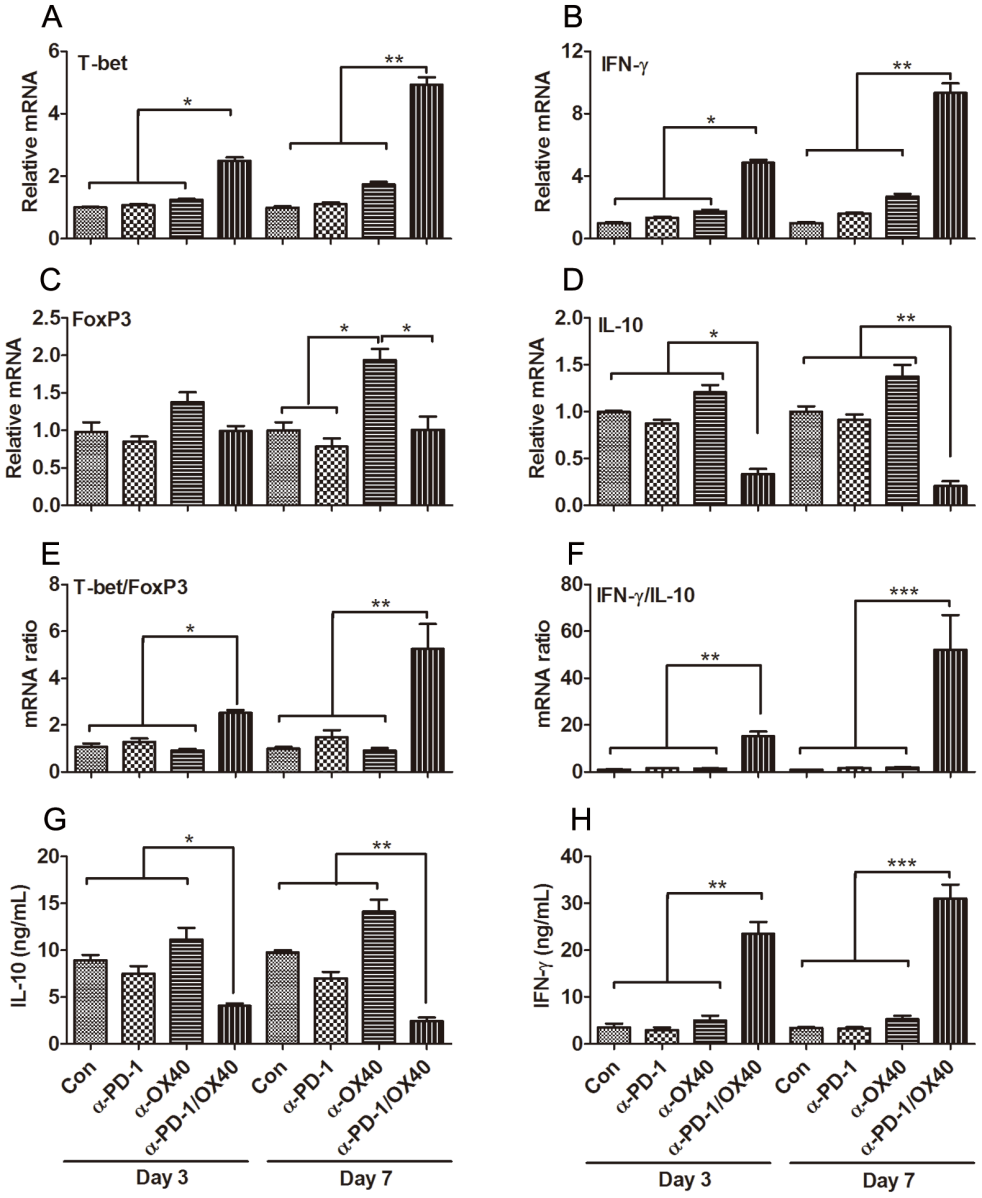
Immune-related gene expression in PIC from treated mice. Mice (5 mice per group) injected i.p. with 1×10^6^ ID8 cells 10 day earlier were injected thrice at 4 days interval with 200 µg of control, single or combined anti-PD-1/OX40 mAb. Three or seven days later, the expression of Th1-associated T-bet and IFN-γ genes and immunoregulatory FoxP3 and IL-10 genes in PIC from treated mice was analyzed by quantitative RT-PCR. The expression of T-bet, IFN-γ, FoxP3 and IL-10 genes are shown in A, B, C and D respectively. The ratios of both T-bet/FoxP3 and IFN-γ/IL-10 are shown in E and F respectively. Freshly isolated PIC from treated mice were stimulated with 50 ng/ml PMA and 1 µg/ml ionomycin for 6 hours (as described in Materials and Methods) and supernatants were assessed for levels of IL-10 (G) and IFN-γ (H). Data were expressed as M±SEM of 5 mice and representative of 2 independent experiments, *P<0.05, **P<0.01, ***P<0.001.

To further substantiate the shifting of tumor microenvironment, we next examined the expression of immune-associated genes in freshly isolated PIC on days 3 to 7 after mAb treatment. Using quantitative RT-PCR, factors associated with immune activation (T-bet and IFN-γ) and those associated with immunosuppressive/regulatory activity (FoxP3 and IL-10) were assessed for their relative levels of expression. On day 3 after treatment, at a time when increased frequencies of T effector cells were observed, transcript levels for T-bet and IFN-γ genes were dramatically enhanced in the combined treatment group (Figure 4A, B). In contrast, the gene expression of immunosuppressive cytokine IL-10 was sharply decreased while FoxP3 gene remained unchanged (Figure 4C, D), which was consistent with the change of Treg and MDSC in PIC. The alteration in the expression of T-bet, IFN-γ and IL-10 genes were more pronounced on day 7 after treatment. Based on the ratios of the stimulatory-to-regulatory gene transcripts (ratios of both T-bet/FoxP3 and IFN-γ/IL-10), we concluded that combined treatment fostered a local immuno-activating tumor microenvironment (Figure 4E, F). Single OX40 triggering moderately increased the expression of all four genes, however, no elevation of both T-bet/FoxP3 and IFN-γ/IL-10 ratios was observed.

To ensure that alterations in IFN-γ and IL-10 RNA expression correlated with alterations at the protein level, PIC were isolated on days 3 to 7 after treatment and stimulated in vitro before analysis of IFN-γ and IL-10 secretion levels by ELISA. Notably, PIC isolated 3 and 7 days after initiating combined mAb therapy produced significantly higher levels of IFN-γ protein and lower levels of IL-10 cytokine compared to that harvested from control or single mAb-treated mice at these same time points (Figure 4G, H).

### Combined anti-PD-1/OX40 mAb treatment elicited an antigen-specific CTL response

**Figure 5:**
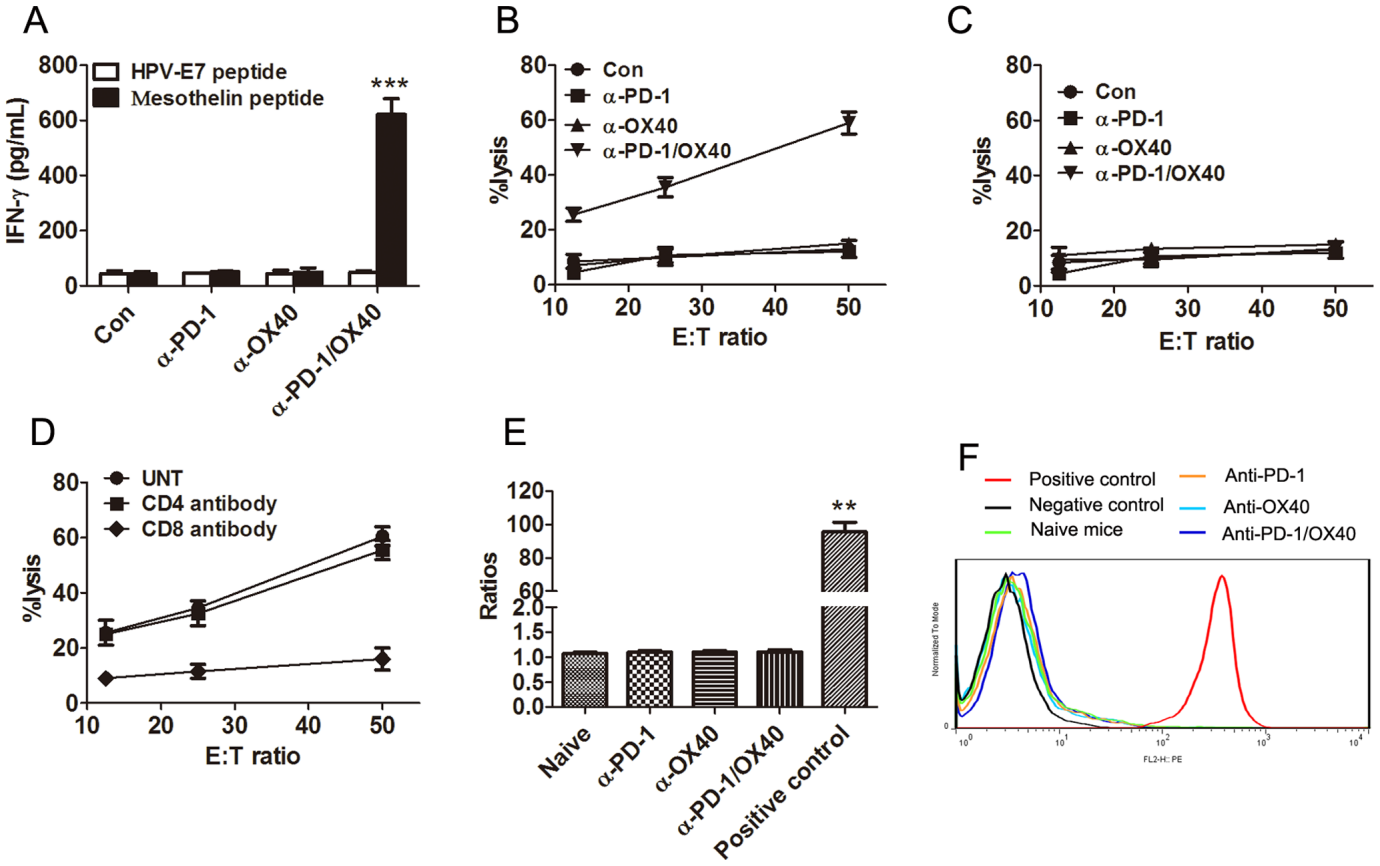
Mice treated with anti-PD-1/OX40 mAbs developed a tumor antigen-specific CTL response. A, Mice injected i.p. with 1×10^6^ ID8 cells 10 day earlier were injected thrice at 4 days interval with 200 µg of control, single or combined anti-PD-1/OX40 mAb. Seven days after the last mAb injection, splenocytes from treated mice were cultured in the presence or absence of H-2Db-restricted mesothelin-derived peptides or control HPV-E7-derived peptide for 3 days and IFN-γ production in the supernatants were assayed by ELISA. B, Pooled splenocytes (5×10^6^) from all mice treated with control, single or combined anti-PD-1/OX40 mAb were incubated with 5×10^5^ UV-irradiated ID8 cells for 4 days prior to subject to analysis of antigen-specific CTL activity by CytoTox 96 Non-radioactive cytotoxicity assay using EL4 cells pulsed with H-2Db-restricted mesothelin peptide as target cells. C, As a specific control, pooled splenocytes were tested cytotoxicity against EL4 target cells pulsed with control HPV-E7 peptide. D, The killing activity of splenocytes from combined mAb treated mice on mesothelin-pulsed EL4 cells was also evaluated in the presence of blocking anti-CD4, anti-CD8 or control antibody. Data were expressed as M±SEM of triplicate wells in A-D. E, Sera were obtained from naïve mice (5 mice), single mAb treated mice (5 mice each group) or 2 mAb treated long-term surviving mice (4 mice). The presence of mesothelin-specific antibodies was determined by flow cytometry via staining mesothelin-expressing ID8 cells with collected sera in a 1∶200 dilution, followed by staining with secondary PE-conjugated anti-mouse IgG antibody and MFI was calculated. Staining with commercially available anti-mesothelin antibody plus secondary antibody or secondary antibody alone was used as positive or negative control. The ratios of MFI from positive control or sera to MFI from negative control were shown. F, A representative histogram in E was shown.

As ID8 cells express the mesothelin, a well-characterized tumor antigen [38], we harvested splenocytes from treated mice, and the same number of splenocytes was cultured in the presence of 10 µg/mL of H-2Db-restricted mesothelin-specific peptide (amino acid 406–414) or control HPV-E7 peptide (amino acids 49–57) for 3 days and analyzed IFN-γ production in culture supernatants by ELISA. Compared with that from control or single mAb-treated mice, splenocytes from combined mAb-treated mice produced significantly higher levels of IFN-γ when stimulated with the mesothelin peptide (P<0.01; Figure 5A). No increased IFN-γ secretion were observed in splenocytes from all groups of mice when they were stimulated with control HPV peptide, demonstrating the induction of mesothelin-specific immune response in mice receiving combined mAb treatment. We further evaluated the cytolytic activity of splenocytes from control or combined mAb treated mice. Splenocytes were restimulated with UV-irradiated ID8 cells for 4 days before CTL assays were performed using EL4 cells pulsed with mesothelin or HPV-E7 derived peptide as target cells. As shown in Figure 5B and 5C, splenocytes from anti-PD-1/OX40 treated mice exhibited significantly higher levels of cytotoxic activity against EL4 cell pulsed with mesothelin but not with HPV-E7 peptide. No specific lysis was seen from the splenocytes from control or single mAb treated mice against either mesothelin or HPV-E7 peptide pulsed EL4 cells. Pre-incubation with CD8 antibody suppressed the cytolytic activity of spleen cells from 2 mAb-treated mice (Figure 5D) and we conclude that combined mAb treatment elicited a tumor antigen-specific CTL response mediated by CD8^+^ T cells. We further determined the induction of mesothelin-specific antibodies in sera from treated mice by flow cytometry. Compared with sera from naïve mice, we did not see an increase of mean fluorescence intensity (MFI) from sera from single mAb treated mice or those long-term survivors receiving combined mAb therapy previously (Figure 5E and 5F), suggesting the absence of mesothelin-specific antibodies in these mice.

## Discussion

The antitumor effect of immunotherapy remains insufficient to achieve long-lasting clinical responses in patients with advanced OC. Here, we demonstrate that combined anti-PD-1/OX40 mAb inhibited the tumor development in the 10-day established ID8 ovarian cancer model, resulting in the long-lasting survival of 60% of mice while individual mAb was ineffective in tumor protection. The findings provide evidence that combined PD-1 blockade and OX40 activation may serve as a novel immunotherapeutic option for treatment of ovarian cancer.

We further sought to understand mechanisms underlying the increased antitumor efficacy by simultaneously removing a major brake on expansion of effector T cells via blockade of the negative regulator PD-1, while at the same time actively driving proliferation, survival, cytokine production and memory formation of T cells through activation of the co-stimulatory receptor OX40. In this setting, we observed that single PD-1 blockade had little effects on tumor-resident CD4^+^ and CD8^+^ T cells while slightly diminishing the levels of immunosuppressive CD4^+^FoxP3^+^ Treg and CD11b^+^GR-1^+^ MDSC in peritoneal cavity. Consistent with previous studies [30], [39], [40], single OX40 triggering significantly promoted the accumulation of CD4^+^FoxP3^+^ Treg and mildly increased the frequencies of peritoneal CD4^+^ and CD8^+^ T cells. Incorporating the decreasing effect of PD-1 blockade on the immunosuppressive Treg and MDSC and promoting effect of OX40 triggering on the effector CD4^+^ and CD8^+^ T cells, combined anti-PD-1/OX40 mAb treatment significantly enhanced the accumulation of peritoneal effector CD4^+^ and CD8^+^ T cells with concomitantly attenuating Treg and MDSC, giving rise to a favorable ratio of the effector T cells to the immunosuppressive cells which is closely related with the effective immunotherapy as previously stated [41], [42]. Consistent with the roles of OX40 and PD-1 in memory T-cell formation [23], [43], individual PD-1 blockade or OX40 triggering modestly increased the percentage of CD44^+^CD62L^−^ effector/memory and/or CD44^+^CD62L^+^ central memory T cells; importantly, combined anti-PD-1/OX40 mAb synergistically promoted the development of effector/memory and central memory T cells in peritoneal cavity, which constitutes the basis for the resistance of long-term surviving mice from 2 mAb treated group to the same tumor rechallenge.

The shift of the immunosuppressive milieu to the immunostimulatory microenvironment by anti-PD-1/OX40 treatment is further corroborated by the increased ratios of both T-bet/FoxP3 and IFN-γ/IL-10 determined by RT-PCR in PIC. ELISA results further confirmed the IFN-γ/IL-10 expression at the protein level. Combined mAb treatment significantly increased the expression of T-bet and IFN-γ genes indicative of Th1-type immune response while attenuating the expression of IL-10 genes indicative of Th2-type immune response, and this expression pattern was also confirmed by ELISA at the protein level, suggesting the shaping of tumor-destructive Th1-type immune response locally by 2 mAb treatment. The data are also concordant with recent exploration of OC immunotherapy by immunoregulatory mAbs (anti-PD-1, anti-CD137, anti-CTLA4 etc) demonstrating the pivotal role of Th1-type immune biasing in antitumor effects [35], [44].

Noticeably, we detected a systemic antigen-specific CTL response to mesothelin expressed on ID8 cells in anti-PD-1/OX40 mAb treated mice, as evidenced by increased mesothelin-specific IFN-γ production and cytolysis by CD8^+^ T cells from these mice. It should be noted that we also observed an increased percentage of splenic CD8^+^ T cells in combined mAb-treated mice compared with that in control or single mAb-treated (9.6±3.4%, 10.1±2.8%, 10.6±3.0% or 12.9±2.4% for control, anti-PD-1 or anti-OX40 or anti-PD-1/OX40 group); even after normalization to the percentage of splenic CD8^+^ T cells, we still saw a significantly increased mesothelin-specific IFN-γ production from combined treated mice (data not shown). As an endogenous non-mutated antigen, mesothelin should be naturally tolerized against; therefore, the induction of mesothelin-specific CTL response by anti-PD-1/OX40 mAb treatment indicates that endogenous tolerance to mesothelin was overcome, which is consistent with previous studies showing the presence of mesothelin-specific immune response in patients with cancers expressing high level of mesothelin [45], [46]. Further work is needed to define whether increased mesothelin-specific CTL response is due to increased precursor frequency of mesothelin-specific CD8^+^ T cells induced by combined treatment. We did not detect mesothelin-specific antibodies in sera from the long-term surviving mice receiving combined mAb treatment by flow cytometry, indicating the absence of mesothelin-specific antibodies or low levels of antibodies undetectable by the current methods.

The importance of CD8^+^ T-cell mediated CTL response was further supported by lymphocyte depletion experiments where the removal of CD8^+^ T cells thoroughly abrogated the antitumor effect of anti-PD-1/OX40 mAb. The data are consistent with previous studies testing the immunotherapies using individual or combined anti-OX40 mAbs demonstrating the pivotal role of tumor-resident CD8^+^ T cells in antitumor effects [29], [32]. The depletion of CD4^+^ T cells also diminished the antitumor effect, indicating an important role of CD4^+^ helper T cells in tumor protection by 2 mAb treatment considering concomitant removal of expanded Treg by anti-CD4 depleting antibody and the key role of Treg in suppressing antitumor immunity [47], [48]. Further studies are needed to provide a deeper insight into the role of effector CD4^+^ T in this setting.

We did not detect the expression of PD-L1/2 on ID8 tumor cells, which may partially explain the refractoriness of ID8 tumor to anti-PD-1 mAb alone as the findings from clinical trials show the correlation between PD-L1 expression on the tumor with the response to anti-PD-1 mAb [20]. Currently, it remains elusive why the addition of anti-OX40 mAb overcomes the resistance of ID8 tumor to anti-PD-1 mAb. Possibly, anti-OX40 mAb treatment may upregulate the expression of PD-L1 on tumor cells and also that of PD-1 expression on the corresponding T cells as described previously for anti-CD137 antibody [49], providing the target for anti-PD-1 mAb and the basis for synergy between anti-PD-1 and anti-OX40 mAb. Further studies are needed to get a deeper insight into the molecular mechanisms underlying this synergistic antitumor effect by anti-PD-1/OX40 mAb. Additionally, we still saw tumor development in 40% of mice receiving 2 mAb treatment with a slower growth dynamics, indicating the occurrence of a “escape” phenotype in tumor cells from these mice, such as loss of MHC class I and/or immunogenic antigens [50]; although we did not found alteration in MHC-I molecule expression on ID8 tumor cells at day 3 and 7 after last treatment (data not show), it is likely that the molecular changes involving “escape” phenotype occurred on a later time point since these mice survived a much longer time. Further studies are warranted to figure out what's happened to tumor cells in these mice.

Currently, a clinical grade anti-OX40 mAb is now being developed and tested [51]. A mouse anti-human OX40 mAb has shown activity in non-human primates with induction of enlarged lymph nodes and spleens and increased T-cell responses [52]. Further evaluation of this mAb in phase I clinical trials shows the evidence for a minimal toxicity while some tumor size reduction and antigen-specific T proliferation and activation were observed [51], [53]. In addition, blockade of PD-1/PD-L1 pathway using anit-PD-1 or anti-PD-L1 mAb in the clinic has shown promising results for the treatment of advanced solid tumors with manageable autoimmune adverse effects. In view of the encouraging results of immunomodulatory mAb in clinic for treatment of multiple solid tumor [54], our finding that PD-1 blockade and OX40 activation synergistically induce a potent antitumor effect in a highly clinical relevant ID8 ovarian cancer model should aid the design of future trials for ovarian cancer immunotherapy.

## Supporting Information

Figure S1 **Representative dotplots showing the purity of peritoneal immune cells before or after isolation using centrifugation via mouse lymphocyte isolation buffer.** Upper and bottom panels denote the representative dotplots of peritoneal lavage before (pre-isolation) and after (post-isolation) isolation by flow cytometric analysis of CD45 expression respectively.
(TIF)
