# Correction: PD-1 Blockade and OX40 Triggering Synergistically Protects against Tumor Growth in a Murine Model of Ovarian Cancer

**DOI:** 10.1371/journal.pone.0186965

**Published:** 2017-10-18

**Authors:** Zhiqiang Guo, Xin Wang, Dali Cheng, Zhijun Xia, Meng Luan, Shulan Zhang

There is an error in the first sentence of the “Monoclonal Antibodies (mAbs)” section within the Methods. The anti-PD-1 antibody should be (Clone RMP1-14; Catalog#: BE0146).
