# Supplementary figures and images for: PD-1 Blockade and OX40 Triggering Synergistically Protects against Tumor Growth in a Murine Model of Ovarian Cancer
Source: PLoS One. 2014 Feb 27;9(2):e89350. doi: 10.1371/journal.pone.0089350 (PMC3937343; doi:10.1371/journal.pone.0089350)

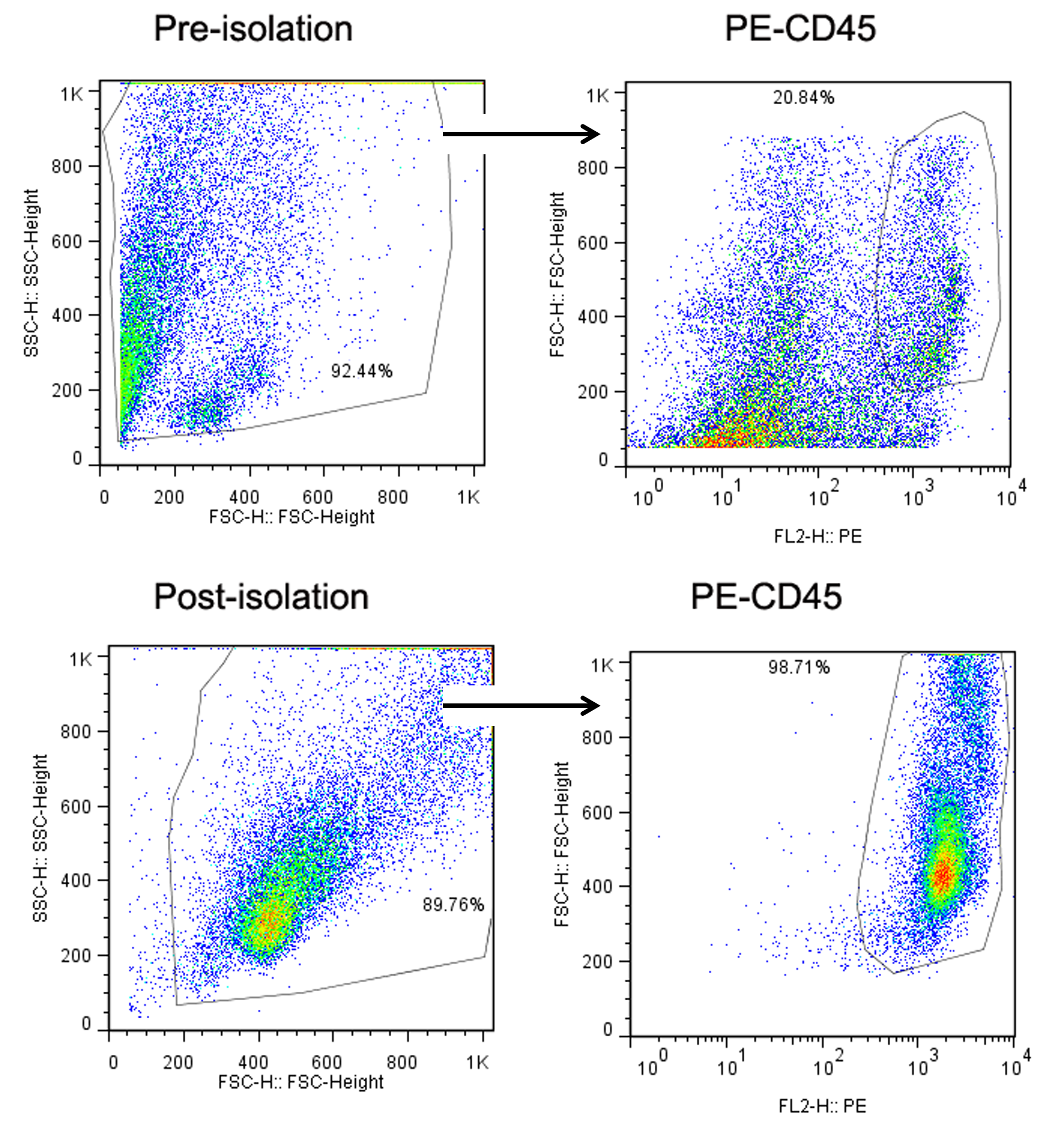

Supplement: Figure S1 — Representative dotplots showing the purity of peritoneal immune cells before or after isolation using centrifugation via mouse lymphocyte isolation buffer. Upper and bottom panels denote the representative dotplots of peritoneal lavage before (pre-isolation) and after (post-isolation) isolation by flow cytometric analysis of CD45 expression respectively. (TIF) [file pone.0089350.s001.tif]
